# Relationships, Current Issues, Safety and Efficacy of Oral Anticoagulation in Cancer Patients with Atrial Fibrillation

**DOI:** 10.3390/jcm12206559

**Published:** 2023-10-16

**Authors:** Antonio Gabriele Franchina, Matteo Rocchetti, Elena Sala, Alessandra Laricchia, Alessandro Minardi, Andrea Spangaro, Marco Guazzi, Stefano Lucreziotti, Alberto Cereda

**Affiliations:** 1 Division of Cardiology, Cardio-Thoracic Department, San Carlo Borromeo Hospital (ASST Santi Paolo e Carlo), Via Pio II n.3, 20153 Milan, Italy; matteo.rocchetti@unimi.it (M.R.); elena.sala2@unimi.it (E.S.); laricchia.a@gmail.com (A.L.); alessandro.minardi@unimi.it (A.M.); andrea.spangaro@unimi.it (A.S.); stefano.lucreziotti@asst-santipaolocarlo.it (S.L.); alberto.cereda@asst-santipaolocarlo.it (A.C.); 2 Division of Cardiology, Cardio-Thoracic Department, San Paolo Hospital (ASST Santi Paolo e Carlo), Via Antonio di Rudinì, 8, 20142 Milan, Italy; marco.guazzi@asst-santipaolocarlo.it

**Keywords:** atrial fibrillation, bleeding, cancer, direct oral anticoagulants, ischemic stroke, systemic embolism, vitamin K antagonists (VKAs)

## Abstract

A relationship between malignancy and impaired hemostasis has been proven, and balancing clotting and bleeding risks can be challenging. Half of cancer patients with atrial fibrillation (AF) do not receive any oral anticoagulation (OAC). Using PubMed on the relationship between cancer and AF and their association with hemostasis, targeting studies comparing vitamin K antagonists (VKAs) and direct OAC (DOAC) strategies in AF cancer patients, three RCTs (>3000 patients) and eight observational studies (>250,000 patients) comparing different OACs were retrieved. The VKA prescribed was always warfarin. Dabigatran was the only DOAC not analyzed in the RCTs but the most used in non-randomized studies, whereas edoxaban-treated patients were the majority in the RCTs. Overall, the DOAC patients showed similar or lower rates of efficacy (thromboembolic) and safety (bleeding) outcomes compared to the VKA patients. DOACs are subject to fewer interactions with antineoplastic agents. DOACs may be preferable to VKAs as a thromboembolic prophylaxis in cancer patients with non-valvular AF.

## 1. Introduction

Worldwide, atrial fibrillation (AF) is the most commonly sustained cardiac arrhythmia in the general population, with an estimated prevalence in 2010 of 2.7–6.1 million in the United States and 6.5–12.3 million in over 55 adults in the European Union [1]. A further increase in prevalence is expected [2,3], related to extended longevity in the general population (advancing age is the most prominent risk factor for AF) [4] and the intensifying search for undiagnosed AF.

Similarly, along with the increasing life expectancy, an incremental trend in cancer prevalence is expected in the coming years, especially lung, colorectal, liver, stomach, and breast cancers [5].

The prevalence of both AF and cancer increases with life expectancy, and some associations have been found between these two pathologies. Cancer patients seem to be at higher risk of developing AF, and older age and systemic disease processes could be involved in this association, as well as the use of cardio-toxic antineoplastic agents [6,7]. Additionally, cancer diagnosis is more common among new-onset AF patients [8].

A relationship between thrombotic state and malignancy has been well recognized for a long time, as described by Trousseau in 1872, who concluded that “spontaneous coagulation is common in cancerous patients”. Clinical and pathological data suggest the systemic activation of the coagulation cascade in patients with cancer—particularly of the pancreas, lung, and gastrointestinal tract [9]—highlighting the prothrombotic potential due to the overexpression of procoagulants and thrombin via the tumor tissue [10]. Conversely, bleeding is common in cancer patients due to local tumor invasion, abnormal tumor vasculature, systemic effects, and anti-tumor treatments (such as radiation therapy, chemotherapy, and immunotherapy) [11]. Moreover, cancer patients have an increased bleeding risk when treated with anticoagulation, especially among subjects with gastrointestinal malignancies [12].

Balancing the increased and competing risks of clotting and bleeding in these patients can be difficult. Thus, anticoagulation therapy management becomes challenging in this population. Accordingly, nearly half of all cancer patients with AF do not receive any anticoagulant therapy [13].

This review aims to summarize the complex relationships among cancer, hemostasis, and AF and show the current evidence for the efficacy and safety of the different types of oral anticoagulants (OACs) in AF patients affected by cancer by looking at the pharmacological interactions with antineoplastic agents and retrieving all studies comparing the use of direct oral anticoagulants (DOACs) versus vitamin K antagonists (VKAs).

## 2. Management of Atrial Fibrillation: A Comparison of Current International Recommendations

Both the European Society of Cardiology (ESC) and the American Heart Association (AHA)/American College of Cardiology (ACC)/Heart Rhythm Society (HRS) rely on the CHA_2_DS_2_-VASc score to guide antithrombotic treatment for the prevention of thromboembolic events [14,15], whereas the Canadian Cardiovascular Society (CCS)/Canadian Heart Rhythm Society (CHRS) employs an algorithm called CHADS-65, based on the patient’s age and their CHADS_2_ risk score [16].

Data from the Danish healthcare system registry show that patients with recent cancer were linked with a higher risk of stroke/thromboembolism at 2 years, even among those with a 0/1 CHADS-VASc score. Therefore, in active malignancy, the decision for anticoagulation should take into consideration the underlying augmented thrombotic risk. Consequently, according to the ESC 2022 guidelines on “cardio-oncology”, anticoagulation may be considered (class of recommendation IIb) in AF patients with CHADS-VASc score of 0 (male) or 1 (female) [17].

According to the guidelines, OAC therapy should not be offered to patients at low risk for stroke (CHA_2_DS_2_-VASc score of 0 in men and ≤1 in women), while it should be considered for patients at intermediate risks (CHA_2_DS_2_-VASc score of 1 in men and 2 in women), with classes of recommendation IIa and IIb according to the ESC and AHA/ACC/HRS, respectively. Anticoagulation treatment is mandatory (class I) in high-thromboembolic-risk patients (CHA_2_DS_2_-VASc score ≥ 2 in men and ≥3 in women) [14,15]. The European Hearth Rhythm Association (EHRA) consensus suggests starting anticoagulation similarly to the ESC guidelines [18]. According to the CCS/CHRS guidelines, anticoagulants are recommended for patients ≥ 65 years old. For younger patients, the decision is based on their CHADS_2_-score evaluation, presenting an OAC indication for subjects with at least one stroke, “CHADS_2_ risk factors” [16].

Aspirin remains an alternative option for American societies for patients with a non-sex-related CHA_2_DS_2_-VASc score of 1 [14,19]. Conversely, European guidelines do not consider aspirin in stroke prevention, while CCS/CHRS recommendations approve antiplatelet therapy exclusively for AF patients < 65 years old with concomitant coronary or vascular disease but no other CHADS_2_ risk factors [16]. While VKAs are recommended for valvular AF, overall guidelines recommend DOACs to prevent stroke in patients with non-valvular AF [14,15,16,18].

Guidelines suggest the use of the HAS-BLED score to determine the individual bleeding risk [15]. However, a high bleeding risk (i.e., HAS-BLED ≥ 3) should not lead to the interruption of oral anticoagulation, as the net clinical benefit of this therapy is even greater amongst such patients. Conversely, modifiable bleeding risk factors should be managed first, and bleeding risk should be reassessed (ESC: Class I) [15]. 

Furthermore, thrombocytopenia is considered an important limitation of the use of antithrombotic drugs or fibrinolytic agents. In cancer patients, thrombocytopenia may be a consequence of chemotherapy toxicity or direct bone marrow infiltration [20]. The risk of bleeding seems to be inversely related to the platelet count, with a severe increase if platelet values are <25 × 109/L [21]. Conversely, mild to moderate thrombocytopenia does not protect from thrombotic events. Therefore, cancer patients remain at risk of venous and arterial thrombosis despite thrombocytopenia [22].

Both the latest ESC guidelines on cardio-oncology and atrial fibrillation consider the closure of the left atrial appendage in patients with contraindication to oral anticoagulation (class of recommendation IIb) [15,17]. This indication is also shared by both the 2021 and 2023 European consensus documents. The 2020 European consensus document and the latest state-of-the-art review on left atrial appendage closure suggest considering this intervention, alternatively to long-term OAC, in the case of contraindication to anticoagulation such as in cancer patients due to high bleeding risk [23,24].

## 3. Cancer and Hemostasis

**Figure 1:**
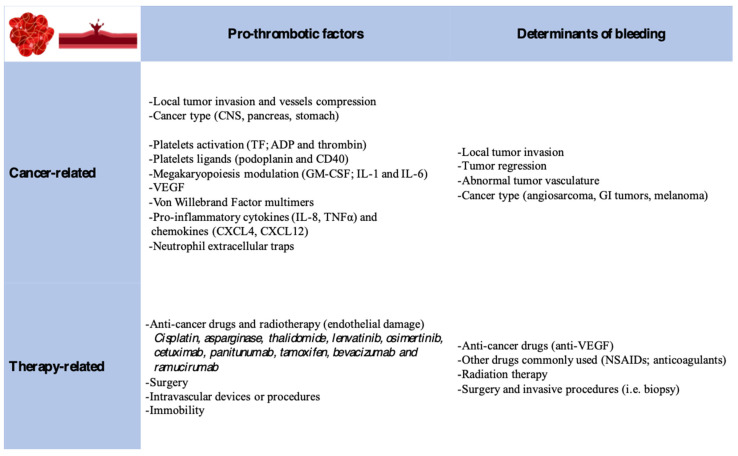
Cancer and therapy-related factors responsible for hemostasis disruption, favoring thrombosis and/or bleeding.

A complex interplay between hemostasis and cancer is driven by a hypercoagulable state often associated with bleeding complications. Indeed, several cancer-related and treatment-related factors flow in this direction (Figure 1).

A pro-thrombotic milieu is favored by patient-related, cancer-related, and treatment-related factors. Cancer-associated factors are linked to the Virchow triad: blood hypercoagulability (release of pro-coagulants and inflammatory cytokines), endothelial damage (tumor invasion, chemotherapy, and intravascular devices or procedures), and venous stasis (extrinsic tumor compression and immobility) [25].

Cancers activate the hemostatic pathway via tissue factor production, ADP and thrombin (platelet activation proteins), and the expression of podoplanin and CD40 (platelets ligands). Tumors also secrete GM-CSF and interleukin-1 and -6 (megakaryopoiesis modulators), which play an important role in thrombus formation. VEGF, IL-1β, and IL-6, via the activation of E-selectin and P-selectin and the secretion of von Willebrand Factor multimers, promote the formation of micro-thrombi. Activated platelets and endothelium can stimulate immune cell proliferation via the production of pro-inflammatory cytokines (IL-8/TNFα) and chemokines (CXCL4/CXCL12) [26].

Chemotherapies (cisplatin, asparaginase, and thalidomide) induce direct vascular endothelial cell activation, the exposure of sub-endothelial TF, and damage signal releases (reactive oxygen species), triggering immune cells propagating the hyper-coagulant status [26].

Molecular target therapies (anitumuma, anitumumab, cetuximab, and anitumumab), anti-hormonal therapy (tamoxifen), and anti-angiogenesis monoclonal antibodies (bevacizumab, ramucirumab) have been associated with hypercoagulability [27,28].

Pancreatic, stomach, and central nervous system cancer have the highest risk of thrombotic formation, whereas lung, ovarian, testicular, and urothelial cancers have intermediate risk; multiple myeloma and aggressive lymphomas show the greatest risk among hematological malignancies [25].

Bleeding is also a common complication of cancer. It can be caused by cancer itself secondary to the local tumor invasion, tumor regression, or abnormal tumor vasculature. Radiation therapy or chemotherapy can also increase bleeding risk. Furthermore, bevacizumab, non-steroidal anti-inflammatory drugs, and anticoagulants that are routinely used in cancer patients can exacerbate the bleeding risk. Finally, thrombocytopenia is commonly observed in oncological patients [11]. Overall, colorectal, prostate, and lung cancers carry the highest bleeding risk [29].

Another contributing factor is the hyperactivity of immature platelet precursors, which are usually released after post-nadir chemotherapy toxicity. Despite the higher risk of bleeding complications associated with low platelet count and dysfunction cancer, patients are still at a higher risk of thromboembolic events. This prompts the need for a higher level of attention when prescribing anticoagulation in cancer patients, with the need to carefully weigh the risks and benefits [30,31].

Cancer patients have a higher risk of thromboembolic events, which, regardless of the presence of AF, concerns the deep venous system/pulmonary embolism as well [32]. In this subset of patients, the therapy of choice requires anticoagulation prolonged after the initial 3 months [33].

The best anticoagulation therapy for pulmonary embolism in cancer patients is still debated; three big RCTs have been recently published regarding the use of direct oral anticoagulation drugs [34,35,36], which have demonstrated a good safety and efficacy profile. The detailed description of these trials and the general indication and current opinion regarding anticoagulation in pulmonary embolism are beyond the scope of this manuscript. 

## 4. Cancer and Atrial Fibrillation

Several studies have shown a link between cancer and an increased risk of new-onset AF. A systematic review and meta-analysis including 5,889,234 subjects documented that solid cancer patients are at higher risk of developing AF compared to non-cancer patients, with the highest risk within 90 days of cancer diagnosis [7]. Conversely, a systematic review and meta-analysis of six studies, including 533,514 participants, showed that new-onset AF was associated with an increased incidence of cancer diagnosis [8]. Similarly, another systematic review and meta-analysis of three prospective studies comprising 390,479 subjects found an increasing risk of subsequent diagnosis of colorectal or breast cancer in patients with AF, concluding that shared risk factors such as old age or systemic diseases may explain this association [6].

A recent nationwide population-based study has linked malignancy types to AF. Overall, cancer (any type) represented an independent risk factor for incident AF during a median follow-up of 4.5 years (Hazard Ratio [HR] 1.63; 95% Confidence Interval [CI] 1.61–1.66) even after adjusting for cardiovascular risk factors. Multiple myeloma patients carried the highest arrhythmic risk in the hematologic malignancy group. When compared to the general population, myeloma patients had a >3-fold risk of AF after 1 year of cancer diagnosis (HR 3.12, CI 95% 2.73–3.57) and at 4.5-year median follow-up (HR 3.34, CI 95% 2.98–3.75). Non-Hodgkin’s lymphoma and leukemia showed strong correlations with AF from cancer diagnosis (respectively, adjusted sub-distribution HR 2.29, 95% CI 2.10–2.51 and HR 2.64, 95% CI 2.38–2.92). In the solid malignancy group, esophageal cancer demonstrated the strongest correlation at both 1-year (HR 2.24, 95% CI 2.00–2.51) and 4.5-year median follow-ups (HR 2.69, CI 95% 2.45–2.95), followed by central nervous system cancer (HR 2.62, 95% CI 2.35–2.91) and lung cancer (HR 2.39, 95% CI 2.30–2.48). Gastric cancers showed the weakest correlation (HR 1.27, 95% CI 1.23–1.32) [37].

Surgical and medical cancer treatments increase the risk of AF [11,28]. Perioperative AF is quite common in patients undergoing thoracic surgery [38]. A long list of anti-neoplastic drugs is linked to new-onset AF, some belonging to older chemotherapy drugs, such as alkylating agents (i.e., cisplatin, cyclophosphamide, and ifosfamide), anthracyclines, and antimetabolites; others are part of newly developed therapeutic strategies such as ibrutinib, immune checkpoint inhibitors, and monoclonal antibodies [39,40].

Furthermore, cancer and AF share common pathophysiological features. Inflammation plays a central role in the development and progression of both AF and cancer. The two diseases also share many common risk factors, such as obesity, smoking, diabetes mellitus, and atherosclerosis [37,38].

## 5. Studies Comparing VKA-Based and DOAC-Based Strategies in Atrial Fibrillation Patients with Cancer

**Figure 2:**
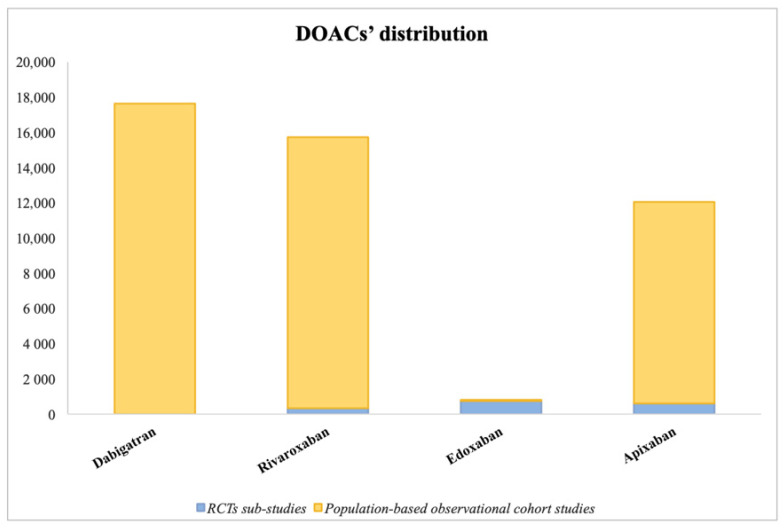
Histogram illustrating the extent of use of each direct oral anticoagulant (DOACs’ distribution) across the various studies comparing anticoagulants.

The management of anticoagulation therapies in patients with AF and cancer comorbidity is quite complex. The most recent data show that often, cancer patients are not initiated with anticoagulants even when bleeding risk status would allow it (HAS-BLED score), leading to an under-treatment of their arrhythmia due to the lack of specific trials and dedicated risk scores in cancer patients and because of their elevated bleeding risk [41]. According to real-life data, up to 44% of AF patients suffering from cancer are not prescribed anticoagulants [13], leading these patients to a risk of thromboembolic consequences. In this review, a summary of studies on OAC therapy in this patient setting, from both Randomized Clinical Trials (RCTs) and Observational/Registry-based Studies, is presented below and illustrated in Table 1, Table 2 and Table 3, while Figure 2 shows the extent of the use of each DOAC across all comparison studies.

### 5.1. Post Hoc Analysis from Main Randomized Clinical Trials Comparing DOACs and VKAs

Four main trials introduced the use of DOACs in clinical practice for patients suffering from non-valvular AF [42,43,44,45]. Three out of the four molecules were then evaluated in separate post hoc analyses investigating patients with a malignancy history: edoxaban, rivaroxaban, and apixaban [46,47,48] (Table 1).

**Table 1:** Randomized clinical trials comparing DOAC-based and VKA-based therapies in atrial fibrillation patients with cancer.

Authors, Year (Ref.)	Type of Study	No. Patients; Randomization	Median Follow-Up (Months)		OACs Evaluated	Primary Outcomes	Conclusion
**Randomized Clinical Trials**							
Fanola C.L. et al, 2022 [46]	ENGAGE AF TIMI 48 trial: sub-study in cancer patients	1153; 1:1:1	33.6	High dose Edoxaban (60 mg qd) or low-dose Edoxaban (30 mg qd) vs. Warfarin		Efficacy outcome: composite of stroke (ischemic or hemorrhagic) or SE	EDOXABAN (both 60 mg and 30 mg) not inferior to Warfarin in efficacy and safety outcomes
						Safety outcome: major bleeding according to ISTH	
Chen S.T. et al., 2019 [47]	ROCKET AF trial: sub-study in cancer patients	640; 1:1	22.8	Rivaroxaban 20 mg qd (or 15 mg QD) vs. Warfarin		Efficacy outcome: composite of stroke or SE	RIVAROXABAN vs. Warfarin: no significant differences in efficacy or safety outcomes
						Safety outcome: composite of major and NMCRB events	
Melloni C. et al, 2017 [48]	ARISTOTLE trial: sub-study in cancer patients	1236; 1:1	21.6	Apixaban 5 mg bid (or 2.5 mg bid) vs. Warfarin		Efficacy outcome: composite of stroke or SE	APIXABAN vs. Warfarin: no significant differences in efficacy or safety outcomes
						Safety outcome: major bleeding according to ISTH	

Abbreviations. bid, bis in die (twice daily); DOAC, direct oral anticoagulant; ISTH, International Society on Thrombosis and Hemostasis; NMCRB, non-major clinically relevant bleeding; OAC, oral anticoagulant; qd, quaque die (once daily); SE, systemic embolism; VKA, vitamin K antagonist.

In the Effective Anticoagulation with Factor Xa Next Generation in Atrial Fibrillation–Thrombolysis in Myocardial Infarction 48 (ENGAGE AF-TIMI 48) sub-study, Fanola CL et al. compared 1153 patients who developed a new or recurrent malignancy and were taking either edoxaban 30 mg (31.9% of patients) or edoxaban 60 mg (33.8%) vs. warfarin (34.3%). Patients were mostly male (68.9%), with a median age of 75 (IQR 68–79) years and a mean CHA_2_DS_2_-VASc score of 4.4 ± 1.3. The most represented cancer was gastrointestinal (20.5%), and metastatic stage accounted for 1% of the total population. The primary efficacy endpoint was the composite of ischemic or hemorrhagic stroke or systemic embolism (SE). The safety endpoint was major bleeding as defined by the International Society on Thrombosis and Hemostasis (ISTH). Edoxaban 60 mg and 30 mg were both non-inferior to warfarin when comparing primary efficacy and safety outcomes in the 2.8 median years’ follow-up. Cancer patients met the primary efficacy endpoint of 1.4%/year in the edoxaban 60 mg group vs. 2.4%/year in the warfarin group (HR 0.60; 95% CI 0.31–1.15). Differences in the rate of major bleeding were not statistically significant between the edoxaban and warfarin group (7.9%/year vs. 8.2%/year; HR 0.98, 95% CI 0.69–1.40). Similarly, in the reduced dose group (edoxaban 30 mg), when compared to warfarin, both primary efficacy and safety endpoints showed the non-inferiority of the DOAC therapy (respectively: 2.04%/year vs. 2.38%/year, HR 0.87, 95% CI 0.47–1.59; and 5.95%/year vs. 8.18%/year, HR 0.73, 95% CI 0.40–1.07) [46].

Both the efficacy and safety of rivaroxaban compared to warfarin showed non-inferiority in AF patients with cancer, as demonstrated in the sub-study of Rivaroxaban Once Daily Oral Direct Factor Xa Inhibition Compared with Vitamin K Antagonism for Prevention of Stroke and Embolism Trial in Atrial Fibrillation (ROCKET-AF) of Chen ST et al. A total of 640 cancer patients (rivaroxaban group: 309; warfarin group: 331) were considered for analyses followed for a median time of 1.9 years. Subjects had a median age of 77 years and a mean CHADS_2_ score of 3.5 ± 1.0. Compared to warfarin, rivaroxaban did not significantly in primary efficacy outcome (rates of stroke/SE) with 1.36 events/100 patient years (8 events) in the rivaroxaban arm and 2.71 (16 events) in the warfarin group (HR 0.52, 95% CI 0.22–1.21). Safety endpoint included major bleeding or clinically relevant non-major bleeding (CRNMB): the rivaroxaban group showed 26.63 events/100 patient years with 97 total events. Similarly, the warfarin arm presented 21.59 events/100 patient years with 96 total events (HR 1.09, 95% CI 0.82–1.44) [47].

The Apixaban for Reduction in Stroke and Other Thromboembolic Events in Atrial Fibrillation (ARISTOTLE) sub-study of Melloni C et al. compared events in AF-cancer patients treated with apixaban or warfarin during a median follow-up of 1.8 years. The authors investigated 1236 patients for the primary composite efficacy outcome of stroke/SE and the primary safety outcome of major bleeding (ISTH criteria). Patients with a history of remote cancer and active cancer had similar median age (75 and 74 years) and mean CHA_2_DS_2_-VASc score of (3.8 ± 1.42 and 3.6 ± 1.52). The effect of apixaban for the prevention of stroke/SE was comparable to warfarin (event/100 patient years: 1.4 vs. 1.2; HR 1.09, 95% CI 0.53–2.26). ISTH major bleeding in apixaban and warfarin groups accounted for 24 events (2.4 events per 100 patient years) and 32 events (3.2 events per 100 patient years), respectively (HR 0.76, 95% CI 0.45–1.29). Apixaban significantly reduced the risk of any bleeding, both majors and minors (204 [26.5] vs. 245 [32.2]) (HR 0.83; 95% CI 0.69–0.99) [48].

### 5.2. Observational Studies and Registry-Based Data on the Comparison between Different OAC Drugs

Table 2 summarizes results from eight population-based cohort studies comparing the safety and efficacy of DOAC vs. VKA in more than 250,000 patients with non-valvular AF and cancer [49,50,51,52,53,54,55,56].

**Table 2:** Not randomized comparative observational studies comparing DOAC-based and VKA-based therapies in atrial fibrillation patients with cancer (for a total amount of 257,339 patients).

Authors, Year (Ref)	Type of Study	No. Patients	Median Follow-Up (Months)	OACs Evaluated	Primary Outcomes	Conclusion
**Observational Not-Randomized Studies**						
Kim K. et al., 2018 [49]	Prospective cohort study	776	1.8	(I) Rivaroxaban or Dabigatran or Apixaban vs. Warfarin	Efficacy outcome: ischemic stroke or SE, all-cause death	(I) DOACs showed lower incidences of efficacy and safety endpoints compared to Warfarin
				(II) Rivaroxaban vs. Apixaban vs. Dabigatran	Safety outcome: major bleeding	(II) No DOAC showed superiority to the other
Sawant A.C. et al., 2019 [50]	Registry-based retrospective study	196,521	12	Apixaban, Rivaroxaban or Dabigatran vs Warfarin	Efficacy outcome: ischemic stroke	DOACs showed similar rates of efficacy endpoint but lower rates of safety endpoints compared to Warfarin
					Safety outcome: hemorrhagic stroke	
Shah S. et al., 2018 [51]	Retrospective study	16,096	11	(I) Rivaroxaban or Dabigatran or Apixaban vs. Warfarin	Efficacy outcome: ischemic stroke Safety outcome: major bleeding	(I) Rivaroxaban and Apixaban showed similar incidences of efficacy and safety endpoints compared to Warfarin, whereas Apixaban showed a similar incidence of efficacy endpoint but a lower rate of safety endpoints
				(II) Dabigatran vs. Rivaroxaban		(II) Rivaroxaban showed lower rates of efficacy endpoint compared to Dabigatran
				(III) Apixaban vs. Rivaroxaban		(III) Apixaban showed lower rates of safety endpoints but not efficacy endpoints than Rivaroxaban
Wu VC. et al., 2020 [52]	Registry-based retrospective study	933	12	Any DOAC (Dabigatran or Rivaroxaban or Edoxaban or Apixaban) vs. Warfarin	Efficacy outcome: ischemic stroke, SE, AMI, and death from any cause	DOACs showed lower rates of efficacy and safety endpoint compared to Warfarin
					Safety outcome: major bleeding, GI bleeding, and intracranial hemorrhages	
Yasui T. et al., 2019 [53]	Retrospective study	224	39.3	Any DOAC (Edoxaban or Dabigatran or Apixaban or Rivaroxaban) vs. Warfarin	Efficacy outcome: ischemic stroke or SE	DOACs showed similar rates of efficacy and safety endpoints compared to Warfarin
					Safety outcome: major bleeding	
Potter AS. et al., 2022 [54]	Retrospective study	390	31.4	Apixaban or Rivaroxaban or Dabigatran vs. Warfarin	Efficacy outcome: cerebrovascular accident	DOACs showed similar rates of ischemic and bleeding endpoints compared to Warfarin
					Safety outcome: intracranial hemorrhage or GI bleeding	
Deitelzweig S. et al., 2021 [55]	Retrospective study	40,271	6–8	(I) Apixaban or Rivaroxaban or Dabigatran vs. Warfarin	Efficacy outcome: stroke/SE	(I) Apixaban showed a lower incidence of efficacy and safety endpoints vs. Warfarin, whereas Dabigatran and Rivaroxaban showed similar efficacy and safety endpoints than Warfarin
				(II) Apixaban vs. Dabigatran		(II) Apixaban showed a lower incidence of efficacy endpoint and a similar incidence of safety endpoint compared to Dabigatran
				(III) Apixaban vs. Rivaroxaban	Safety outcome: major bleeding	(III) Apixaban showed a similar incidence of both efficacy and safety endpoints compared to Rivaroxaban
				(IV) Rivaroxaban vs. Dabigatran		(IV) Rivaroxaban showed a similar incidence of efficacy endpoint but a higher risk of safety endpoint compared to Dabigatran
Ording AG. et al., 2021 [56]	Retrospective study	2128	12	Any DOAC (Apixaban, Rivaroxaban, Dabigatran or Edoxaban) vs. Warfarin	Efficacy outcome: NA	DOACs and Warfarin showed the same rates of GI bleeding
					Safety outcome: GI bleeding	

Abbreviations. AMI, acute myocardial infarction; DOAC, direct oral anticoagulant; GI, gastrointestinal; OAC, oral anticoagulant; SE, systemic embolism; VKA, vitamin K antagonist.

The largest study included 196,521 patients [50], while the smallest included 224 patients [54]. Five studies did not include edoxaban [49,50,51,54,55], and consequently, it was the least represented DOAC among these cohort studies, while the most represented was apixaban (Figure 2). Four authors performed a propensity score (PS) matched analysis paring warfarin patients with similar DOAC patients [51,52,54,55]. Two articles also performed DOAC–DOAC comparisons for primary safety and efficacy outcomes [49,51,55].

Kim K. et al. published a prospective cohort study on 1651 cancer patients taking OAC. The mean age was 74.2 ± 8.3 years for the DOACs-treated patients vs. 67.5 ± 8.0 years for the warfarin-treated patients (*p* < 0.001), with a higher mean CHA_2_DS_2_-VASc in DOACs group than in VKA group (3.8 ± 1.7 vs. 3.4 ± 1.4; *p* < 0.001). The median follow-up was 1.8 years. The authors performed a PS matching-based analysis with 388 warfarin patients coupled with 388 DOAC patients, of which 138 (35.6%) patients were treated with apixaban, 110 (28.3%) with rivaroxaban, and 140 (36.1%) with dabigatran; no patient was treated with edoxaban. Stomach cancer was the most common malignancy (20.9% of DOAC patients, 20.4% in the warfarin group), and metastatic cancer accounted for 53 (13.7%) patients in the DOAC group and 45 (11.6%) patients of warfarin group. The main efficacy outcome was a composite of ischemic stroke/SE. The safety primary outcome was represented by ISTH major bleeding. The rate of ischemic stroke/SE was 1.3 vs. 5.9 events per 100 patient years (*p*-value < 0.001), while the rate of major bleeding was 1.2% vs. 5.1%/year (*p* < 0.001) in the DOACs vs. warfarin group, respectively. However, when dividing warfarin patients with time to therapeutic range (TTR) ≥ 60% and TTR < 60%, efficacy and safety endpoints remained significantly lower in DOAC patients compared to the TTR < 60% warfarin subgroup (*p* < 0.01). Compared with the TTR ≥ 60% subgroup, DOAC patients showed a similar rate of ischemic stroke but a significantly lower rate of major bleeding (*p* = 0.03). Lastly, when comparing full vs. reduced doses of DOACs, the incidence rates of ischemic stroke/SE and major bleeding did not significantly differ. Similarly, no significant interaction was observed according to DOAC type [49]. 

In the study of Sawant AC et al., 196,521 patients with active cancer and AF and a mean age of 76 years were selected. The most common DOAC used was dabigatran (7.6% of the total study population), followed by rivaroxaban (6%) and apixaban (8%), while all other patients were treated with warfarin. The primary endpoint was represented by one-year all-cause mortality, which was higher in the VKA group than in the DOAC population (44.9% vs. 26.2%, *p* < 0.001). The rates of ischemic and hemorrhagic strokes were gathered as well: the warfarin population had a similar risk of ischemic stroke (13.5%) compared to patients receiving dabigatran (11.1%), rivaroxaban (12.0%), or apixaban (14.0%), but patients treated with warfarin had higher rate of hemorrhagic stroke events (1.2% vs. 0.6% vs. 0.7% vs. 0.8%) [50].

In the paper from Shah S et al., 16,096 patients with active cancer who initiated OAC were selected. A PS was used to control for confounding via matching or adjustment. The warfarin group included 10,021 patients (62.3%), while the most commonly used DOAC was rivaroxaban (17.4% of the total study population), followed by dabigatran (13.6%) and apixaban (6.7%). Warfarin patients had a mean age of 75.4 years and were slightly older than their DOAC counterparts (mean age of 74.0 years). Warfarin patients had a higher CHA_2_DS_2_-VASc score (4.6) compared to the DOACs group (4.2). The mean study follow-up was 12 months. The most common cancer type was breast cancer (21.4% of rivaroxaban patients, 20.8% of dabigatran patients, 23.4% of apixaban patients, and 17.8% of warfarin patients). The primary outcome was severe bleeding events (intracranial hemorrhage (ICH) or gastrointestinal bleeding), while secondary outcomes included other bleeding events, ischemic stroke, and venous thromboembolism (VTE). All three DOACs showed a similar rate of ischemic stroke when compared to the matched warfarin users: rivaroxaban (HR 0.74, 95% CI 0.40–1.39, *p*-value 0.35), dabigatran (HR 0.89, 95% CI 0.56–1.42, *p*-value 0.63), and apixaban (HR 0.71, 95% CI 0.19–2.60, *p*-value 0.6). Apixaban was the only direct anticoagulant to show a reduction in severe bleeding when compared to warfarin (HR 0.37, 95% CI 0.17–0.79, *p*-value 0.01), whereas both rivaroxaban and dabigatran showed a similar outcome rate compared to warfarin (HR 1.09, 95% CI 0.79–1.50, *p*-value 0.9 vs. HR 0.96, 95% CI 0.72–1.27, *p*-value 0.75, respectively). However, the apixaban group was the smallest one and the one with the shortest follow-up (6 months). In the DOAC–DOAC-matched comparison between dabigatran and rivaroxaban users, dabigatran was associated with a higher rate of ischemic stroke (HR 7.61, 95% CI 1.5–38.1, *p*-value 0.01), while no significant difference was observed regarding severe bleeding rate (HR 1.07, 95% CI 0.5–2.3, *p*-value 0.86). However, rates of VTE were numerically lower in the dabigatran rather than in the rivaroxaban population (*p*-value 0.06). Furthermore, compared with rivaroxaban, apixaban was associated with lower rates of severe bleedings (HR 0.29, 95% CI 0.13–0.65, *p*-value 0.002) and VTE (HR 0.23, 95% CI 0.12–0.47, *p* < 0.0001), while the rates of ischemic stroke were not significantly different (HR 0.52, 95% CI 0.13–2.17, *p*-value 0.37) [51].

In the study by Wu VC et al., after PS-matching, a population of 672 cancer patients treated with warfarin or DOAC for AF were selected, with 336 subjects in each group. 

No data concerning the number of patients prescribed with each DOAC were available. The mean age was 75.08 ± 9.5 years for warfarin patients and 75.09 ± 9.9 years for DOACs group (*p*-value 0.99). There was no difference in the CHA_2_DS_2_-VASc score (4.2 ± 1.89 for warfarin patients, 4.21 ± 2.0 for DOAC patients, *p* = 0.94). No data concerning the type frequency of the cancer or the stage of the disease was available. The main efficacy outcome included ischemic stroke/SE, acute myocardial infarction, and death from any cause. Safety outcomes comprised major bleeding, gastrointestinal bleeding, and ICH. Both results were available at 6 and 12 months. The results showed significant differences in ischemic stroke/SE (HR 0.42, 95% CI 0.24–0.74) and major bleeding (HR 0.26, 95% CI 0.09–0.76) in favor of the DOAC population compared to the warfarin group in one year. Similar results were also observed at 6-month follow-ups for ischemic stroke/SE (HR 0.45, 95% CI 0.25–0.82) and major bleeding (HR 0.21, 95% CI 0.05–0.96). No statistically significant differences were reported in gastrointestinal bleeding, acute myocardial infarction, and death at 6 and 12 months [52].

In the Yasui T et al. article, the authors compared 224 patients with AF and cancer. The most commonly prescribed DOAC was apixaban (36.2% of DOAC patients), followed by rivaroxaban (34.6%), dabigatran (19.7%), and edoxaban (9.4%); 43.3% of patients were prescribed with warfarin. Age and thromboembolic risk factors were similar among the two groups: mean age was 72.7 ± 7.1 and 72.7 ± 7.2 years for the DOACs and warfarin group, respectively (*p*-value 0.99); CHA_2_DS_2_-VASc score was 3.1 ± 1.4 and 3.0 ± 1.5, respectively (*p*-value 0.84). Patients were followed for 1 year. The most common cancers were gastrointestinal (44.2% of the total population), and 22.2% of the population presented metastases. The study outcomes were stroke/SE (efficacy) and ISTH major bleeding (safety). The rates of stroke or SE and major bleeding events were not statistically significantly different between DOACs and warfarin groups: in the DOACs group, three patients suffered from thromboembolic events compared to four patients in the warfarin group (2.8%/year vs. 5.4%/year, *p*-value 0.35), while four patients met the safety outcome both in DOACs and in warfarin groups (4.0%/year vs. 6.5%/year, *p*-value 0.50) [53].

More recently, Potter AS et al. conducted a retrospective cohort study including 1133 patients with active cancer and AF, with a median follow-up of 31.4 months. A total of 291 (25%) patients were treated with warfarin, the remaining 842 (75%) with a DOAC: apixaban (57.2%), rivaroxaban (6.4%), or dabigatran (0.4%; 3 patients). After PS matching, 195 DOAC patients matched with 195 warfarin patients were selected, with a comparable mean age (72.5 ± 8.4 vs. 71.6 ± 9.1 years in the DOAC vs. warfarin group, respectively, *p* = 0.26) and CHA_2_DS_2_-VASc score (3.5 ± 1.8 vs. 3.5 ± 1.6, *p* = 0.67). Among solid malignancies, in terms of cancer types, the genitourinary was the most represented in both populations. The efficacy endpoint was the cumulative incidence of cerebrovascular accidents. Two safety endpoints consisted of ICH and gastrointestinal bleeding, and they were analyzed separately. The authors demonstrated similar risk for cerebrovascular events in the warfarin and DOACs groups (HR: 0.738, 95% CI 0.33–1.63, *p*-value 0.45). Similarly, intracranial and gastrointestinal bleeding rates were not different between warfarin and DOAC groups (respectively: HR 0.295, 95% CI 0.03–2.71, *p*-value 0.28; HR 1.819, 95% CI 0.77–4.28, *p*-value 0.17) [54].

Deitelzweig S et al. proposed a subgroup analysis of the Anticoagulants for Reduction in Stroke: Observational Pooled Analysis on Health Outcomes and Experience of Patients (ARISTOPHANES) study comparing safety and risks of anticoagulant therapies in 40,271 cancer patients. Subjects were divided into four main groups according to the prescribed OAC: warfarin (15,371/38% of the population), apixaban (9517/24%), dabigatran (2742/7%), and rivaroxaban (12,641/31%). Among these patients, the most common cancer type was prostate cancer (29%). The mean age among apixaban, rivaroxaban, dabigatran, and warfarin patients was 77.2 ± 8.3 years, 76.2 ± 8.3, 75.6 ± 8.2 and 77.6 ± 7.9, respectively. The CHA_2_DS_2_-VASc score was distributed as follows: 3.99 ± 1.5 in the apixaban group, 3.84 ± 1.6 in the rivaroxaban group, 3.77 ± 1.6 in the dabigatran group, and 4.22 ± 1.5 among warfarin patients. The mean follow-up of the study ranged from 6 to 8 months. A 1:1 PSM analysis was performed matching DOAC patients with corresponding warfarin patients and DOAC patients with other DOAC patients. Six total comparisons were performed: three DOAC–warfarin comparisons (apixaban–warfarin with 8236 patients for each subgroup, dabigatran–warfarin with 2470 patients each, and rivaroxaban–warfarin with 9988 patients each) and three head-to-head DOAC–DOAC (apixaban–dabigatran with 2413 patients each, apixaban–rivaroxaban with 8608 patients each, and dabigatran–rivaroxaban with 2553 patients each). The primary efficacy endpoint was stroke/SE, whereas major bleedings (gastrointestinal, intracranial, or other major sites hemorrhages) represented the primary safety endpoint. In DOAC–warfarin comparisons, apixaban patients had a lower risk of stroke/SE compared to warfarin (HR 0.59, 95%CI 0.45–0.78), whereas dabigatran and rivaroxaban had no statistically significant lower risks (dabigatran HR 0.88, 95%CI 0.54–1.41; rivaroxaban HR 0.82, 95%CI 0.62–1.08). Even in safety outcomes, the apixaban cohort was the only one presenting lower risk than warfarin patients (HR 0.58, 95% CI 0.50–0.68); rivaroxaban and dabigatran were found to have similar risks of major bleeding compared to warfarin (dabigatran HR 0.76, 95% CI 0.57–1.01; rivaroxaban HR 0.95, 95% CI 0.85–1.06). In the DOAC–DOAC comparisons, apixaban led to a lower risk of stroke/SE than dabigatran (HR 0.41, 95% CI 0.22–0.77), while no significant difference was observed in apixaban–rivaroxaban (HR 0.81, 95% CI 0.60–1.08) and dabigatran–rivaroxaban (HR 0.90, 95% CI 0.50–1.63) comparisons. When considering the primary safety endpoint, apixaban showed a lower safety than rivaroxaban (HR 0.66, 95% CI 0.54–0.80) but similar to dabigatran (HR 0.83, 95% CI 0.58–1.19); dabigatran presented a lower risk of major bleeding when compared to rivaroxaban (HR 0.71, 95% CI 0.52–0.95). Finally, after a further subgroup analysis according to cancer type, no significant interactions were observed between specific treatments and cancer types [55].

Lastly, in 2021, Ording et al. [56]. compared bleedings among gastrointestinal cancer patients of a Danish nationwide cohort study data, including 2128 subjects treated with DOACs (1476 patients; 69%) or VKA (652 patients; 31%) for AF. Apixaban was the most commonly prescribed DOAC (40.9% of patients), followed by rivaroxaban (22.4%), dabigatran (22.4%), and edoxaban (0.9%). The majority of patients suffered from colorectal cancer (86%), and 42% of the total had active cancer. The major endpoint was gastrointestinal bleeding at 1 year. The two anticoagulation strategies had similar rates of bleeding: 5.36 per 100 patients/years for DOAC and 5.62 per 100 patients/years for VKA (HR 0.95, 95% CI 0.63–1.45). The result remained consistent after considering active cancer patients only (HR 1.00, 95% CI 0.53–1.88) [56]. 

### 5.3. Data from Single-Arm Observational Studies

We considered data from two studies regarding patients with AF and a history of cancer treated with DOACs without a statistical comparison between each of them (Table 3).

**Table 3:** Single-arm observational studies on DOACs use in atrial fibrillation patients with cancer.

Authors, Year (Ref)	Type of Study	No. Patients	Median Follow-Up (Months)	OACs Evaluated	Primary Outcomes	Conclusion
Pacholczak-Madej R. et al., 2021 [57]	Prospective cohort study	48	40	Apixaban or Rivaroxaban or Dabigatran	Efficacy outcome: stroke/TIA or SVE	DOACs have shown to be an effective and safe therapeutic option in breast cancer patients with AF during adjuvant hormonal therapy
					Safety outcome: major bleeding and CRNMB	
Laube ES. et al, 2017 [58]	Retrospective study	163	7.3	Rivaroxaban 20 mg qd (or 15 mg QD)	Incidence of stroke, major bleeding, death and CRNMB leading to the discontinuation of Rivaroxaban for at least 7 days	Rates of events are comparable to the results of the ROCKET AF trial

Abbreviations. AF, atrial fibrillation; CRNMB, clinically relevant non-major bleeding; DOAC, direct oral anticoagulant; OAC, oral anti-coagulant; qd, quaque die (once daily); SVE, symptomatic venous thromboembolism; TIA, transient ischemic attack.

The study of Pacholczak-Madej R et al. [57] was a prospective cohort study with a 40-month median follow-up, including 48 women with surgically eradicated breast cancer treated with adjuvant hormonal therapy: 13 received apixaban, 22 rivaroxaban, and 13 dabigatran. One stroke and two CRNMB were observed on apixaban. One transient ischemic attack (TIA), three major bleedings, and two CRNMBs were reported on rivaroxaban. Three VTEs were documented in dabigatran-treated individuals without any bleeding or cerebrovascular events.

In the study of Laube ES et al. [58], information from 163 subjects with active cancer and AF treated with rivaroxaban was retrospectively analyzed. Patients were mainly men (56%) with a mean CHA_2_DS_2_-VASc score of 3.2, mostly with stage IV cancer (59%). The most represented cancer type was lung cancer (19%). During the mean observation period of 175 days, 35 patients reached a clinical endpoint: 2 strokes, 2 major bleedings, 10 CRNMBs, and 21 deaths. All the individuals with stroke or major bleeding had metastatic cancer, with lung/gastrointestinal and gynecological/breast origins for these two outcomes, respectively. No SE events were reported.

## 6. Drug–Drug Interaction between OAC and Anti-Neoplastic Agents

The concomitant presence of AF and cancer exposes patients to interactions between OAC and anti-neoplastic drugs. Although this is a very broad topic, a brief overview of the most relevant interactions between anticoagulants and antineoplastic agents is presented below.

Combined treatments of DOACs appear to have good safety profiles with the majority of chemotherapy drugs [55]. However, DOACs have different pharmacokinetic characteristics due to their interactions with P-Glycoprotein, cytochrome P450 (CYP450), plasmatic protein binding, and renal/hepatic metabolism. The inducers and inhibitors of P-Glycoprotein and CYP450 alter DOAC plasmatic levels, thus influencing their effects [59,60,61,62]. EHRA guidelines guided the co-treatment with DOAC and antineoplastic drugs, as described below [18]. According to these recommendations, more frequent pharmacokinetic interactions with consequent effects on plasma levels are known regarding the concomitant use of apixaban and rivaroxaban with antineoplastic drugs, although in most cases, these interactions are not clinically significant. Vinca alkaloids lead to a mild–moderate decrease of DOACs’ area under the curve (AUC) due to P-Glycoprotein induction and competition with CYP3A4 and their co-assumption requires caution, especially during therapy with vinblastine (strong pharmacokinetic interactions). Paclitaxel and docetaxel are not contraindicated in co-therapy with DOACs, but instability of plasma levels of apixaban and rivaroxaban is possible with the use of paclitaxel (reduced) and docetaxel (increase). DOACs can be usually associated with anthracyclines; only co-treatment with doxorubicin is not advisable because it could mildly reduce DOAC plasma levels due to induction of P-Glycoprotein (strong) and CYP3A4 (mild). Combined treatments of asparaginase with DOACs increase bleeding time, but co-treatment is not contraindicated. Alkylating agents such as cyclophosphamide and ifosfamide are responsible for mild CYP3A4 inhibition and competition, determining the mild increase in plasma levels of apixaban and rivaroxaban, although their co-assumption is not contraindicated. No relevant interactions are known when DOACs are prescribed together with platinum-based agents, methotrexate, or some monoclonal antibodies such as alemtuzumab, cetuximab, rituximab, bevacizumab or trastuzumab. Also, caplacizumab, ipilimumab, and ramucirumab increase bleeding time, but the association with DOACs is possible with careful monitoring. Venetoclax, a BCL-2 inhibitor, could increase the DOACs effect because of the competition and inhibition of P-Glycoprotein and CYP3A4, leading to a possible increase in DOACs’ AUC. Kinase inhibitors such as imatinib and crizotinib strongly inhibit P-Glycoprotein and CYP3A4, significantly increasing the DOACs’ AUC. Thus, their co-treatment is contraindicated. Ibrutinib inhibits P-Glycoprotein and collagen-induced platelet aggregation, increasing bleedings, including ICH; thus, co-treatment DOACs-ibrutinib needs careful monitoring [63,64]. Immune modulating agents such as cyclosporine and tacrolimus have a strong inhibition on P-Glycoprotein and CYP3A4, leading to an increase in DOACs’ AUC [65]. The concomitant use of tacrolimus/cyclosporine and dabigatran is contraindicated, while very caution is needed in association with apixaban, rivaroxaban, and edoxaban. Co-treatment with DOACs and enzalutamide or abiraterone is contraindicated [18,66], while bicalutamide, anastrozole, and tamoxifen could be associated with DOACs with careful monitoring. Finally, the use of oral glucocorticoids increases bleeding risk in patients co-treated with DOACs. 

Due to the wide range of interactions with cytochrome P450, the possibility of drug interaction between VKA and antineoplastic agents is greater than with other anticoagulants [67]. In a 2020 review, attention was focused on the possible fluctuations in the international normalized ratio (INR) that can be caused by interactions between warfarin and antineoplastic drugs, highlighting the necessity of careful INR monitoring during combined therapy [68]. Specifically, 5-fluorouracil (5-FU) has been shown to increase INR [69,70], probably due to interference with the synthesis of hepatic cytochrome P-450 2C9 [71]. Patients should be closely monitored for a possible delayed interaction that may occur with each repeated cycle of 5-FU [72]. Capecitabine, an oral prodrug of 5-FU, enhances the anticoagulant action of warfarin, possibly due to a down-regulation of CYP2C9 by capecitabine or its metabolites [73,74]. Interferon inhibits hepatic microsomal enzymes, resulting in a reduction in warfarin metabolism [75]. Co-administration of warfarin-tamoxifen is contraindicated due to an increase in anticoagulation levels and consequent increased bleeding risk, probably due to the CYP2C9 inhibition [76]. Both warfarin and tyrosine-kinase inhibitors have high protein binding rates (>90%) and that could lead to a competitive effect; however, definitive evidence for drug–drug interactions on protein displacement is lacking [75].

## 7. Conclusions

Several epidemiological and pathogenetic links between AF and cancer are quite established. Despite international guidelines recommending anticoagulation in AF patients at high thromboembolic risk, the coexistence of cancer often leads to under-treatment because of patients’ frailty and difficulties in managing OACs and their interactions with anti-neoplastic agents. Although robust randomized clinical trials are not available to guide anticoagulation in patients with both cancer and AF, data from the literature (both post hoc analysis from RCTs and population-based cohort studies) showed that patients treated with DOACs have similar or lower rates of thromboembolic and bleeding events compared to patients with VKA prescription. Anti-tumor agents have effects on the plasma levels of apixaban and rivaroxaban more than with other DOACs, in most cases with no contraindication; remarkably, VKA is subject to more clinically relevant interactions with antineoplastic drugs than DOACs. Overall, DOACs may be a valid alternative to VKAs for thromboembolic prophylaxis in AF-cancer patients, although only subgroup analyses from RCTs or observational studies are currently available on this topic.

## Abbreviations

(5-FU)	5-Fluorouracil
(AMI)	Acute Myocardial Infarction
(AHA/ACC/HRS)	American Heart Association/American College Of Cardiology/Heart Rhythm Society
(ENGAGE AF-TIMI 48)	Anticoagulation With Factor Xa Next Generation In Atrial Fibrillation–Thrombolysis In Myocardial Infarction 48
(ARISTOTLE)	Apixaban For Reduction In Stroke And Other Thromboembolic Events In Atrial Fibrillation
(AUC)	Area Under The Curve
(AF)	Atrial Fibrillation
(CCS/CHRS)	Canadian Cardiovascular Society/Canadian Heart Rhythm Society
(CRNMB)	Clinically Relevant Non-Major Bleeding
(CI)	Confidence Interval
(CYP)	Cytochrome P
(DOACs)	Direct Oral Anticoagulants
(EHRA)	European Heart Rhythm Association
(ESC)	European Society Of Cardiology
(GI)	Gastrointestinal
(HR)	Hazard Ratio
(INR)	International Normalized Ratio
(ISTH)	International Society On Thrombosis And Hemostasis
(ICH)	Intracranial Hemorrhage
(NMCRB)	Non-Major Clinically Relevant Bleeding
(OACSs)	Oral Anticoagulants
(PS)	Propensity Score
(RCT)	Randomized Clinical Trial
(ROCKET-AF)	Rivaroxaban Once Daily Oral Direct Factor Xa Inhibition Compared With Vitamin K Antagonism for Prevention of Stroke and Embolism Trial in Atrial Fibrillation
(SE)	Systemic Embolism
(TTR)	Time To Therapeutic Range
(TIA)	Transient Ischemic Attack
(VTE)	Venous Thromboembolism
(VKAs)	Vitamin K Antagonists
